# Post-thaw application of ROCK-inhibitors increases cryopreserved T-cell yield†

**DOI:** 10.1039/d3md00378g

**Published:** 2023-09-05

**Authors:** Natalia Gonzalez-Martinez, Matthew I. Gibson

**Affiliations:** a Department of Chemistry, University of Warwick Gibbet Hill Road Coventry CV4 7AL UK m.i.gibson@warwick.ac.uk; b Division of Biomedical Sciences, Warwick Medical School, University of Warwick Gibbet Hill Road Coventry CV4 7AL UK

## Abstract

Emerging cell-based therapies such as CAR-T (Chimeric Antigen Receptor T) cells require cryopreservation to store and deliver intact and viable cells. Conventional cryopreservation formulations use DMSO to mitigate cold-induced damage, but do not address all the biochemical damage mechanisms induced by cold stress, such as programmed cell death (apoptosis). Rho-associated protein kinases (ROCK) are a key component of apoptosis, and their activation contributes to apoptotic blebbing. Here we demonstrate that the ROCK inhibitor fasudil hydrochloride, when supplemented into the thawing medium of T-cells increases the overall yield of healthy cells. Cell yield was highest using 5 or 10% DMSO cryopreservation solutions, with lower DMSO concentrations (2.5%) leading to significant physical damage to the cells. After optimisation, the post-thaw yield of T-cells increased by approximately 20% using this inhibitor, a significant increase in the context of a therapy. Flow cytometry analysis did not show a significant reduction in the relative percentage of cell populations undergoing apoptosis, but there was a small reduction in the 8 hours following thawing. Fasudil also led to a reduction in reactive oxygen species. Addition of fasudil into the cryopreservation solution, followed by dilution (rather than washing) upon thaw also gave a 20% increase in cell yield, demonstrating how this could be deployed in a cell-therapy context, without needing to change clinical thawing routines. Overall, this shows that modulation of post-thaw biochemical pathways which lead to apoptosis (or other degradative pathways) can be effectively targeted as a strategy to increase T-cell yield and function post-thaw.

## Abstract 2

The post-thaw yield of cryopreserved T-cells is shown to be increased by addition of a ROCK inhibitor.

## Graphical abstract

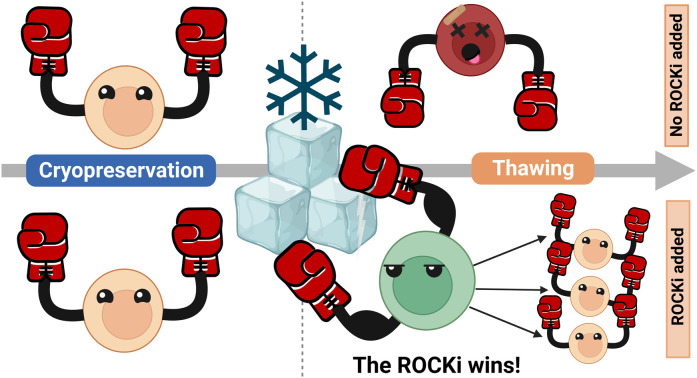

## Introduction

Cell-based therapies deploy living cells to fight disease, often after *ex vivo* modifications.^1^ Chimeric antigen receptor (CAR) T-cell therapy use the body's own immune cells (T-cells) to target cancers after being genetically modified to target tumour antigens.^2^ Since 2017, several CAR T-cell therapies have been approved by the FDA,^3^ with 1270 currently in clinical trials according to https://www.ClinicalTrials.gov. As living cells are used in these therapies, they pose additional manufacturing and storage challenges. It is vital to reduce cellular degradation associated with extended normothermic or hypothermic storage, as the effectiveness of the treatment is dependent on cell viability after infusion to the patient.^4^ The most practical long-term storage and transportation solution is cryopreservation. Cryopreservation involves the storage of cells by cooling typically below −80 °C.^5,6^ Cellular cryopreservation offers many benefits to the accessibility, safety, flexibility in manufacturing and patient administration of these therapies.^7^ For example, if there are long distances between the product manufacturing site and clinic, or to increase product storage time when a patient is currently too unwell to receive the therapy.^6,8^

Cryopreservation requires a tightly controlled process, where cryoprotectants such as dimethyl sulfoxide (DMSO) should be added to the cryopreservation media. This minimises physical damage caused by ice crystals in cellular membranes and organelles, and osmotic pressure due to exposure to a hypertonic environment as extracellular water contents freeze.^5,9^ There has been a large volume of work on the formulation and optimisation of cryopreservation media to maximise cellular outcomes, as well as the development of tools to address biophysical (*i.e.* ice growth) modes of damage. This includes ice binding proteins,^10^ controlled nucleation,^11,12^ ice recrystallisation inhibitors,^13–16^ and macromolecular cryoprotectants.^17–19^ Despite the success of all these additives, they do not target the biochemical damage induced by hypothermic temperatures, nor the damage mechanisms which remain and impair post-thaw function. Hence there is an opportunity to develop approaches inspired by ‘medicinal chemistry’ to improve post-thaw outcomes using drugs targeting these pathways.

Programmed cell death or apoptosis is one of the biochemical pathways that becomes dysregulated following cryopreservation and contributes to cell death in multiple cell types.^20,21^ Cellular exposure to hypothermic temperatures during the freeze–thaw process trigger multiple stress factors, including ionic imbalances, energy deprivation and free radical production, which initiate apoptosis.^22–24^ Cells undergoing apoptosis may appear viable immediately post-thaw, but the number of viable cells decreases 24–48 hours later (often referred to as delayed-onset cell death).^25^ During apoptosis, a number of hallmark events happen such as nuclear condensation, DNA fragmentation, membrane blebbing and phosphatidylserine externalisation.^26,27^ Apoptosis is mediated by the cysteine protease (caspase) cascade signalling system, which can be initiated by either intrinsic or extrinsic factors. The intrinsic pathway is primarily mediated by the mitochondria in response to intracellular stresses while the extrinsic is initiated in the cell membrane due to the activation of death receptors.^22,27^ In addition to these core pathways, there are multiple additional regulators, such as the Rho-associated protein kinases (ROCK). These are cleaved by caspase-3 during apoptosis, leading to the formation of stress fibres and membrane blebs.^28,29^ Specifically, the ROCK II isoform can promote Fas death receptor expression in the cell membrane as a method of apoptotic regulation.^30^ For further information about the apoptosis pathways see here.^31,32^

Immune cells, and particularly T-cells, are susceptible to cryopreservation damage and delayed-onset cell death.^33,34^ Cryopreservation decreases their viability,^35^ functionality,^36,37^ and suitability to perform immune assays due to increased variability.^38,39^ This becomes even more relevant when discussing CAR-T cell therapy cryopreservation, as it is important to ensure that these act efficiently and conserve high viabilities.^40,41^ Studies have shown lower cell viability and cytokine expression,^42^ lower surface marker expression,^43^ upregulation of apoptosis-associated gene expression^7^ and lower cellular expansion after thawing compared to fresh CAR-T products.^44^ Although results vary along studies, their cryopreservation is usually supported, as evidenced by the FDA approval of cryopreserved CAR-T products, which allow their centralised manufacturing.^45–48^

Biochemical pathways dysregulated after cryopreservation will differ between cell types, therefore it is important to approach this in a “cell-type dependent” manner.^49^ The literature suggests that T-cells are affected by apoptosis after cryopreservation, with around 40% of cells undergoing apoptosis 8 h post-thaw, followed by extensive cell death as shown by Sarkar *et al.*^50^ In Jurkat cells (a model T-cell line) inhibition of apoptosis using a pan-caspase inhibitor improved cell recovery and survival.^51^ Peripheral blood mononuclear cells are also affected by apoptosis after cryopreservation, as evidenced by the high percentage of apoptotic T-cells in the DMSO-free formulation developed by Pi *et al.* The authors therefore concluded that targeting apoptosis would be beneficial in future studies.^52^ Other exciting avenues for biochemical preconditioning have been reported in other cell types. One example is the incubation of cells with the osmolyte l-proline for 24 hours prior to freezing. This “primes” cells for cryopreservation by temporarily slowing down cell growth, leading to increased post-thaw recoveries.^53–55^

Here we investigate the application of a ROCK inhibitor to improve the yield and viability of Jurkat cells after cryopreservation. The ROCK inhibitor fasudil hydrochloride was added to the cells immediately upon thawing and was found to improve the yield by up to 20%. The exact incubation time was crucial, as too long, or too short incubation times reduced the benefit in cell recovery. Pre-freeze incubation with fasudil showed a lower but still beneficial cell recovery compared its addition post-thaw. This data demonstrate the benefit of targeting specific biochemical pathways to improve cryopreservation outcomes, which could improve CAR-T cell recovery and therapeutic outcomes.

## Experimental section

### Cell cryopreservation

Cell cryopreservation media consisted of Advanced RPMI 1640 media supplemented with 10% FBS and either 2.5, 5 or 10% DMSO (Sigma), depending on the experiment performed. Before cryopreservation, Jurkat cells were centrifuged at 300 g and resuspended in antibiotic-free cell culture media at a density of approximately 8 × 10^6^ cells mL^−1^. 500 μL of the cell suspension was pipetted into a 2 mL cryovial (Sigma Aldrich), freezing a total of 4 × 10^6^ cells. 500 μL of cryopreservation media (prepared at 2× the final DMSO concentration, *e.g.*, 10% DMSO for a final concentration of 5%) was then added into the cell suspension. Cryovials were then placed in a Cool LX vial freezing container (Corning) and into a −80 °C freezer, to cool at a rate of 1 °C min^−1^. After 24 hours, cells were thawed in a water bath at 37 °C for 2–3 minutes until only a small ice crystal was left, and the cell suspension was diluted in 9 mL of cell culture media, centrifuged at 300*g* for 5 minutes and resuspended in 1 mL cell culture media.

### Addition of fasudil hydrochloride into post-thaw media

After resuspending the cryovial contents in 1 mL of cell culture media, 50 μL of cell suspension was placed into each well of round bottom 96 well plates (Sarstedt). Fasudil hydrochloride (Sigma) solutions were then prepared at 2× the desired concentration in cell culture media and sterile filtered with a 0.22 μm syringe filter (Fisher Scientific). 50 μL was added to each well. The final fasudil concentration used varied from 40 μM to 1.25 μM. Cells were then incubated at 37 °C and 5% CO_2_ for 4 h (timepoint selected after performing optimisation experiments). The plate was subsequently centrifuged at 300*g* and the media was changed to regular cell culture media (see ESI†). Cell health assessments (such as cell recovery) were performed 24 hours post-thaw. The minimum number of technical replicates per condition was 3.

### Cell recovery calculations

24 hours post-thaw, an aliquot of cells was diluted 1 : 1 with 0.4% Trypan blue (Sigma Aldrich). The number of cells with intact membranes (unstained cells) were counted using a haemocytometer (Sigma Aldrich). Cell recovery was calculated by dividing the number of live cells obtained post-thaw against the cell number frozen and expressed as a percentage.

### Fasudil supplementation in cryopreservation media experiments

Two different cryopreservation media were compared in this section: Advanced RPMI 1640 media containing 10% FBS and 5% DMSO, and that same medium supplemented with 50 μM fasudil hydrochloride. DMSO and fasudil were prepared at 2× the concentration, sterile filtered, and kept at 4 °C until use. Jurkat cells were harvested, counted, and resuspended in Advanced RPMI 1640 media supplemented with 10% FBS. The target cell number was 10 × 10^6^ cells mL^−1^. 500 μL of this cell suspension (5 × 10^6^ cells) was then placed into cryovials (Nalgene) followed by 500 μL of the cryopreservation medium. Cryovials were then placed in a Cool LX vial freezing container and into a −80 °C freezer. After 24 hours, cryovials were thawed for 2–3 minutes in a water bath at 37 °C until no ice crystals were visible, and the 1 mL cell suspension was transferred into a 15 mL Falcon tube containing 9 mL of cell culture media. The tube contents were gently mixed and 100 μL of cell suspension was plated into round bottom 96-well plates (Sarstedt), targeting 50 000 cells per well without considering cryopreservation damage. Unfrozen cells were plated at this cell density as a control for metabolic activity assays. For cell recovery studies, cell density before cryopreservation was used to calculate percentage recovery. All cell health assays were performed 24 hours post-thaw.

### Statistical analysis

**Fig. 1:**
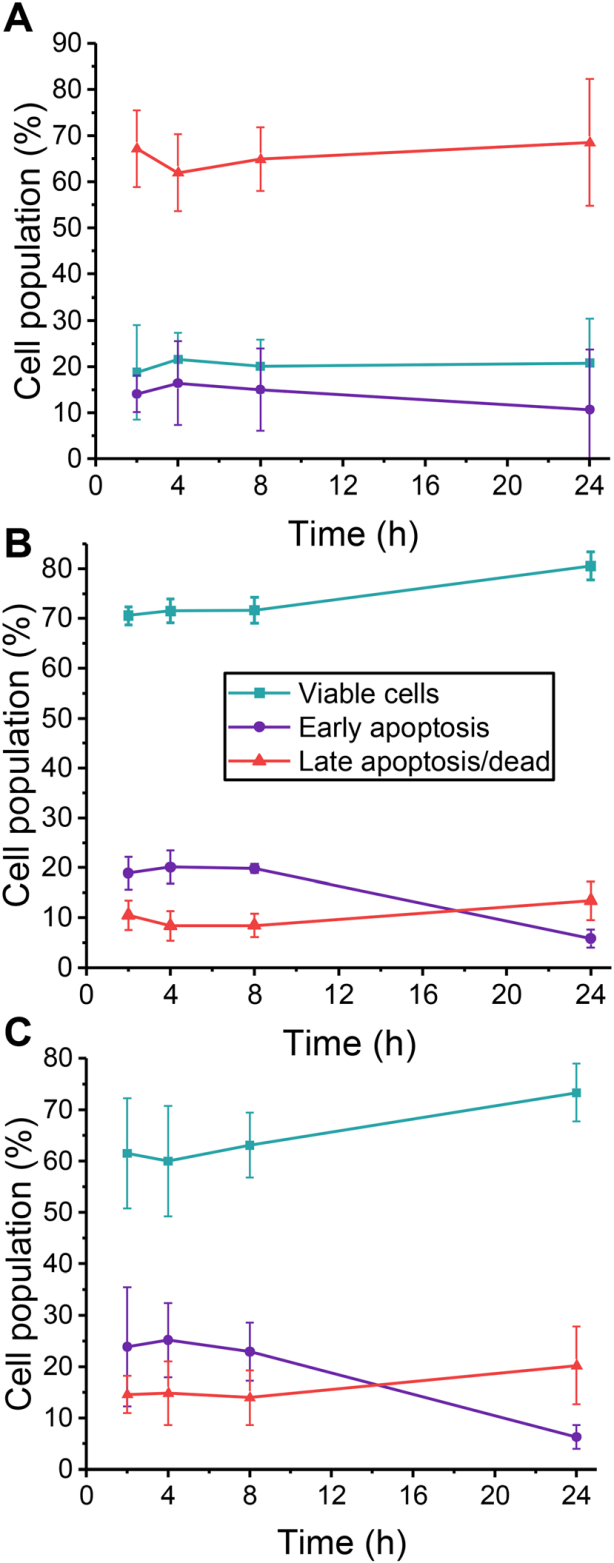
Jurkat cell viability and apoptosis levels as a function of post-thaw culture time. Cells were cryopreserved at a cell density of 4 × 10^6^ cells mL^−1^ in the following DMSO concentrations: (A) 2.5% (v/v) (*n* = 3, 3 technical replicates each); (B) 5% (v/v) (*n* = 4, 3 technical replicates); (C) 10% (v/v) (*n* = 4, 3 technical replicates). Apoptosis was measured by flow cytometry after staining with Annexin V-FITC and propidium iodide (PI). For cell population (%) calculations viable cells were Annexin V-FITC^−^/PI^−^; early apoptotic cells were Annexin V-FITC^+^/PI^−^ and late apoptotic/dead cells were Annexin V-FITC^+^/PI^+^. Data presented as mean ± standard deviation (SD) of *n* independent experiments.

**Fig. 2:**
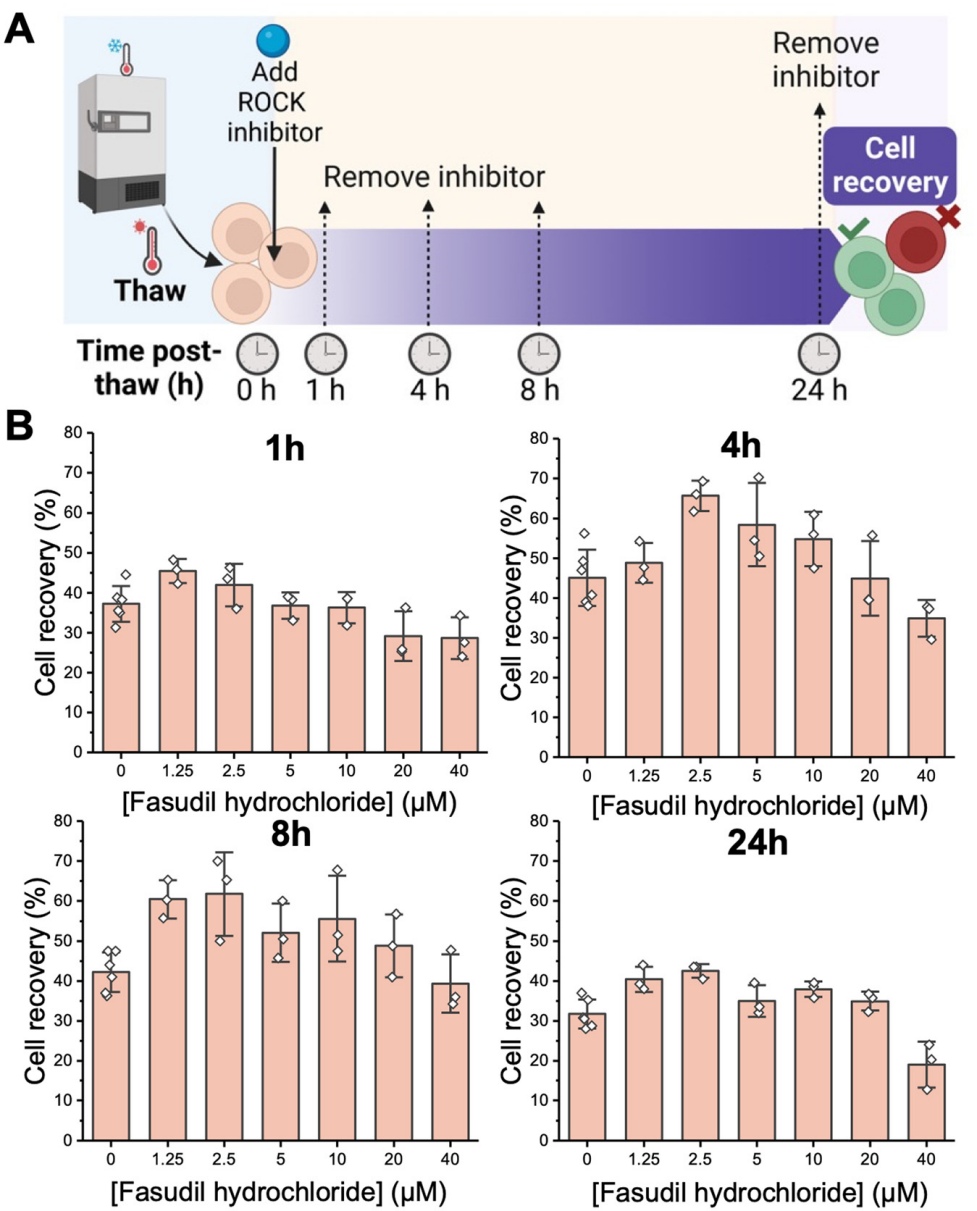
Post-thaw recovery screen as a function of exposure time to fasudil hydrochloride (0–40 μM) after cryopreservation in 10% DMSO. 4 × 10^6^ cells mL^−1^. (A) Schematic of experimental set up; (B) post-thaw (24 h) recovery data (1 independent experiment, ≥3 technical replicates) assessed using the trypan blue assay. Data are presented as mean ± SD of technical replicates and each is taken 24 hours after removal of fasudil. Statistical analysis not performed, as this was an optimisation process and only 1 independent experiment was performed.

**Fig. 3:**
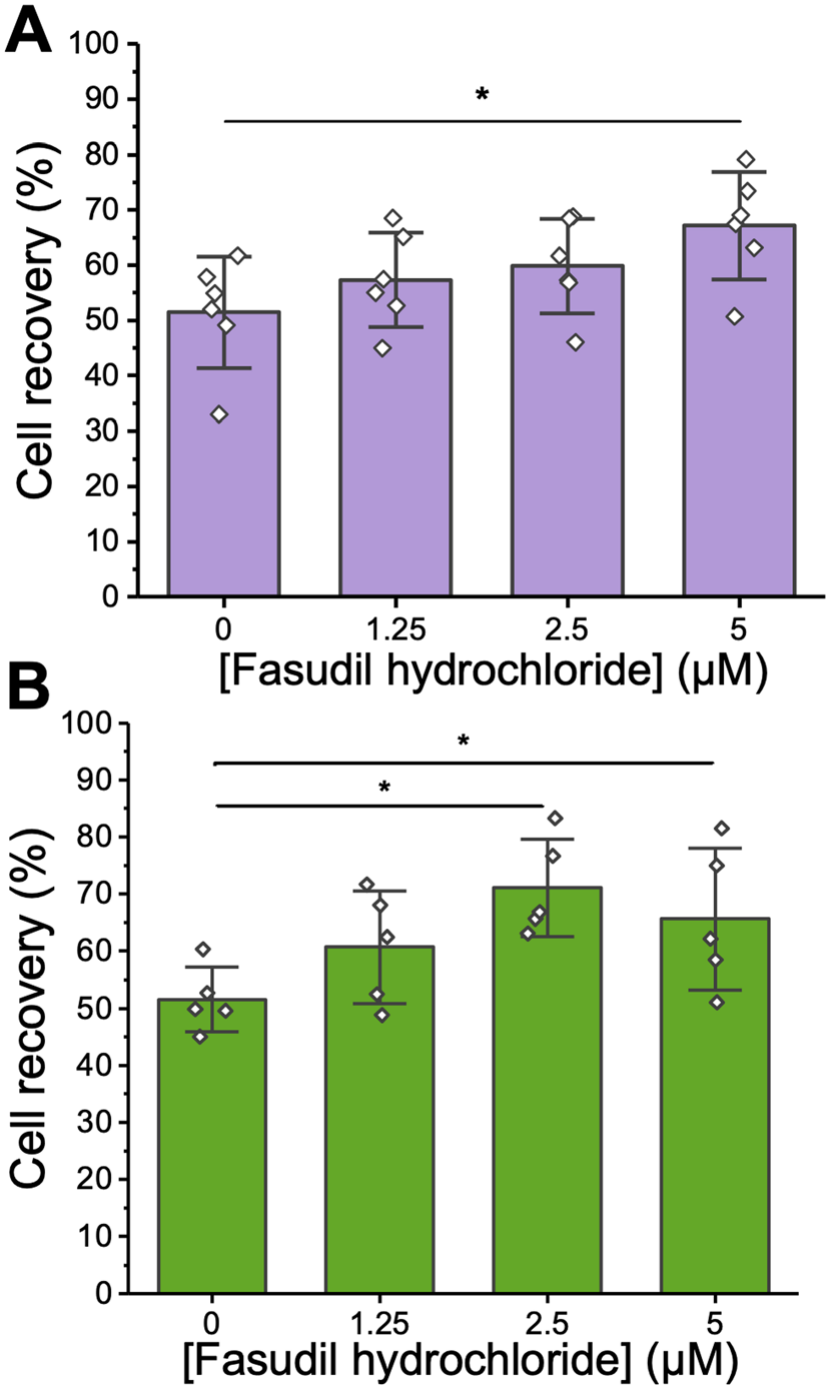
Post-thaw cell recovery as a function of fasudil hydrochloride concentration. Cells were cryopreserved at a cell density of 4 × 10^6^ cells mL^−1^ in either (A) 5% (v/v) DMSO (*n* = 6, 3 technical replicates each) and (B) 10% (v/v) DMSO (*n* = 5, 3 technical replicates each) and both (A and B) were incubated with fasudil for 4 hours after thawing. Cell recovery was assessed 24 hours post-thaw using the trypan blue assay. Data are presented as mean ± SD of *n* independent replicates. Asterisks represent *p* < 0.05 (1-way ANOVA, Tukey *post hoc* test).

**Fig. 4:**
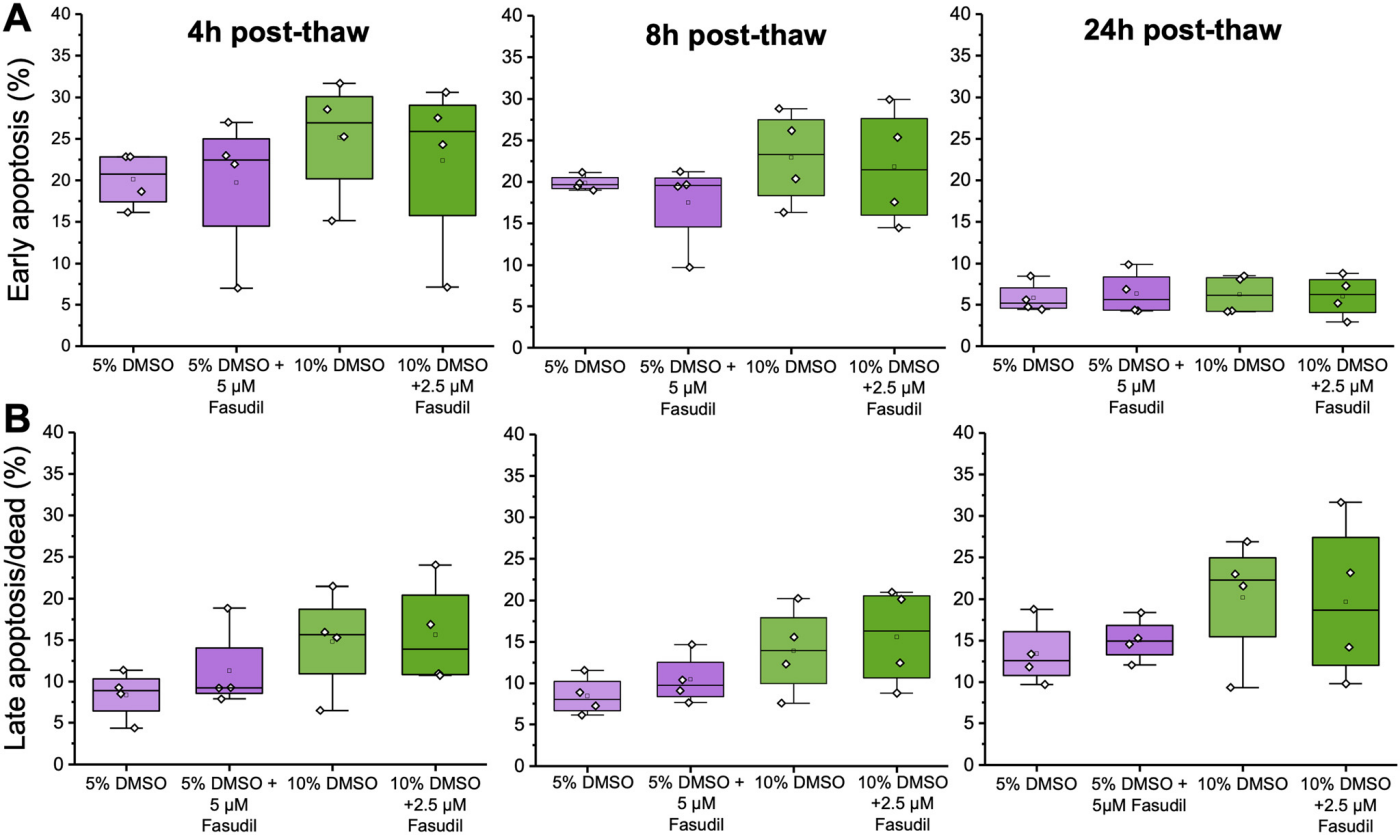
Cryopreservation-induced apoptosis measurements over 24 hours, after freezing with either 5 or 10% DMSO and with or without supplementation of fasudil in post-thaw media. Apoptosis was quantified using Annexin V-FITC and propidium iodide (PI) staining using flow cytometry. (A) Early apoptotic cell percentage (Annexin V-FITC^+^/PI^−^); (B) late apoptotic/dead cells (Annexin V-FITC^+^/PI^+^) the boxplots display the median (black line) and mean cell population percentage (clear square box). The box shows the upper and lower quartiles, the whiskers represent the lowest and highest values obtained (within 1.5 times the inter quartile range). Individual points correspond to each independent repeat (*n* = 4), each representing the average of ≥3 technical repeats.

**Fig. 5:**
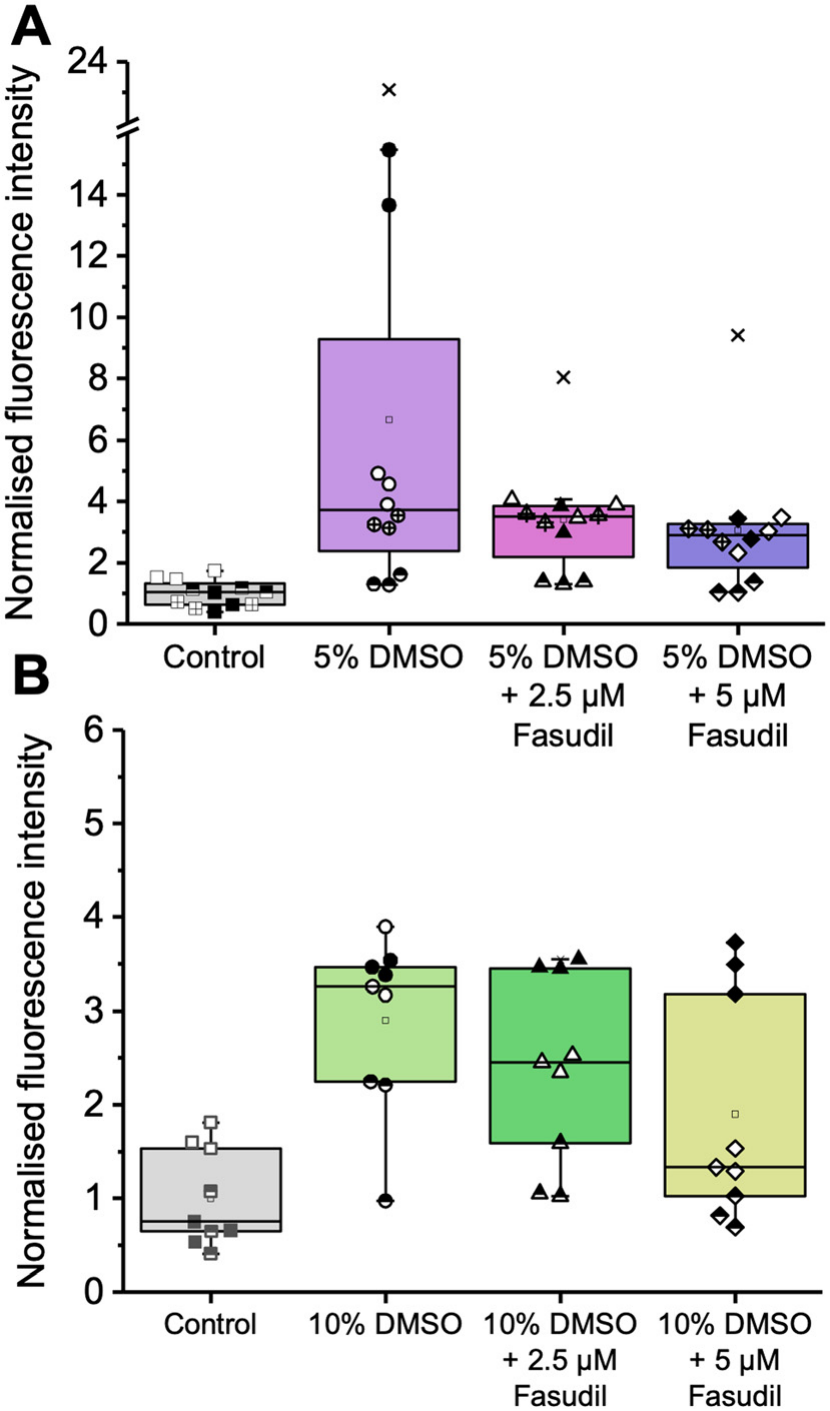
Intracellular ROS production after cryopreservation with (A) 5% DMSO (4 independent experiments with 3 technical replicates) or (B) 10% DMSO (3 independent experiments, 3 technical replicates) at *t* = 24 h post-thaw. Cells were labelled with 1 μM carboxy-H_2_DCFDA for 1 h at 37 °C and analysed using flow cytometry. Results were normalised to the mean of the (median) fluorescence intensity values from the negative controls across experiments. The boxplots display the median fluorescence intensity (black line) and mean (clear square box). The box shows the upper and lower quartiles, the whiskers represent the lowest and highest values obtained (within 1.5 times the inter quartile range) and crosses represent outliers. The boxplot shows all replicates, with different shading styles corresponding to different independent repeats.

**Fig. 6:**
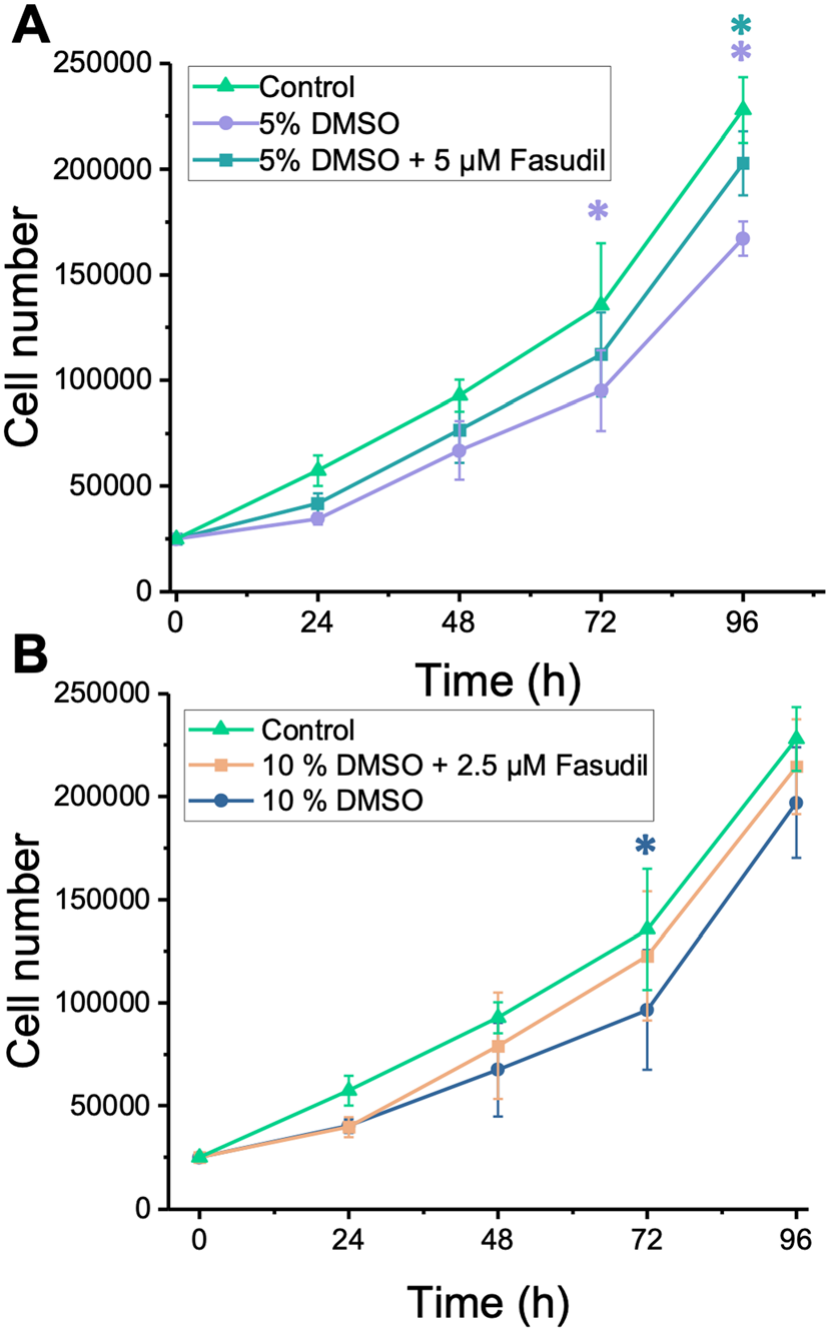
Growth curves of cryopreserved and fresh Jurkat cells over 96 hours. (A) Mean cell number after cryopreservation in 5% DMSO and either left untreated after thawing (circle) or treated with fasudil for 4 h post-thaw (square). 25 000 cells per well were used for all conditions. An unfrozen untreated cell control was included for comparison. (B) Mean cell number after cryopreservation in 10% DMSO and treated as above. All data are presented as mean ± SD of 3 independent repeats with 3 replicates each. In (A) purple asterisks show a significant difference (*p* < 0.05, 2-way repeated measures ANOVA) between the 5% DMSO condition and unfrozen control. The blue asterisk shows a significant difference between experimental groups (untreated *vs.* treated). In (B) blue asterisk shows a significant difference between 10% DMSO and control.

Statistical analysis was performed using Origin (Version 2022). The data was analysed for normality using the Shapiro–Wilk Test, and Levene Test to test for equality of variance between groups. When the distribution and variances were equal between groups, mean comparisons among two or more groups were performed using one-way ANOVA and Tukey's *post hoc* test against the appropriate control and results were reported as mean ± SD, as shown in Fig. 1–3. For growth curve experiments, a 2-way repeated measures ANOVA was performed to compare the effect of treatment (or its absence) and time post-thaw, on cell number (Fig. 6). Results were considered statistically significantly different when *p* < 0.05. When the tests determined a significant difference between the distribution, or equality of variance tests, a non-parametric test (Kruskal–Wallis) was used, together with Dunn *post hoc* test (Fig. 5). Results were visually represented using boxplots, highlighting the median and upper and lower quartiles. Additionally, the Welch *T*-test was used for non-parametric comparison between 2 groups (Fig. 4).

## Results and discussion

To test the hypothesis that fasudil can improve Jurkat cell post-thaw recovery, we first quantified the cell viability and apoptosis levels following cryopreservation. Jurkats were cryopreserved at 4 × 10^6^ cells mL^−1^ in the indicated concentrations of DMSO in a −80 °C freezer for 24 h, below the intracellular glass transition temperature,^56^ which is suitable for short term storage.^57^ Then, from 2 to 24 hours post-thaw, flow cytometry was used to determine the relative fractions of viable, early, and late apoptotic cells (Fig. 1) [Note, the total cell recovery is not reported in this section, but the fraction of recovered cells in each sub-population]. The most significant population of cells after cryopreservation in 2.5% DMSO was late apoptotic/dead cells. This is consistent with previous observations, where low DMSO concentrations significantly damaged the physical integrity of cells due to the freeze/thaw process, resulting in very low recoveries.^58^ Hence, attempting to modulate biochemical pathways after cryopreservation with 2.5% DMSO was not pursued further. In contrast, the cells were mostly intact after freezing with 5 or 10% (v/v) DMSO, with approximately 20% early apoptotic cells at 4 hours post thaw, decreasing to approximately 10% over 24 hours. Fasudil would be expected to reduce the number of early apoptotic cells (and hence decrease late apoptosis over time) if applied immediately post-thaw. Therefore, these data shows that 5 or 10% DMSO conditions could be amenable to rescuing cell recovery by the deployment of a ROCK inhibitor. For subsequent experiments, both DMSO concentrations were assessed to represent “standard” DMSO concentrations typically used and maximise cell recovery.

With the above information to hand, supplementation of fasudil after Jurkat cryopreservation in 10% DMSO was probed. Fasudil was added into the thawing media at a range of concentrations from 0 to 40 μM. It was then removed by replacing the media at the indicated time points, and recovery was measured at 24 hours post-thaw (see Fig. 2A):^25^ this ensures all data was collected after the same post-thaw time, with only the fasudil incubation period varied. Fasudil cytotoxicity was evaluated alongside these experiments, showing no negative effect in cell viability up to 20 μM, with some reduction in viability (to 80%) observed at 40 μM (ESI† Fig. S1). This should be considered when assessing the results. Extended exposure to fasudil for 24 hours led to a limited increase in post-thaw recovery (Fig. 2B), as did 1 hour, presumably due to insufficient time for it to take effect. However, both 4 and 8 hour exposures showed substantial increases in post-thaw cell recovery, with 2.5 μM fasudil giving the largest enhancement of ∼20%. This itself is a significant observation, showing that the modification of the thawing media – rather than cryopreservation conditions – is an easy route to increase cell yields.

The ROCK inhibitor Y-27632 was previously shown to increase survival of embryonic stem cells by reducing apoptosis, as well as aiding cell attachment, which is not relevant to suspension lines, such as Jurkats.^59^ Use of other ROCK inhibitors have been reported.^60,61^ Caspase and oxidative stress inhibitors have also been applied to hematopoietic progenitor cells to increase yields.^21^ In Jurkat or T-cells, there are (to the best of our knowledge) two reports of post-thaw apoptosis mitigation. zVAD-fmk, a pan-caspase (apoptosis) inhibitor which, when added to Jurkats and other cell types for 24 hours post-thaw, led to increased cell numbers. This effect was also observed when supplemented into the cryopreservation media itself, but to a lower magnitude.^51^ In CD4+ T-cells (from rhesus macaques), post-thaw addition of 50 μM zVAD-fmk reduced the early apoptotic population from approximately 20 to 10%.^50^ We are not aware of ROCK inhibitors being deployed for T-cells, but the above examples agree with our observations that targeting specific biochemical pathways (rather than the freezing process) can increase cell yield.

To further explore the benefits of fasudil, the experiments were repeated using both 5 and 10% DMSO, and a focused concentration range of fasudil from 1.25 to 5 μM. In both cases there was a clear increase in post-thaw cell yield upon addition of fasudil into the thawing media (4 hours exposure, yield determined 24 hours post thaw). After cryopreservation in 5% DMSO, only the supplementation of 5 μM fasudil significantly increased cell recovery. When using 10% DMSO, both 2.5 and 5 μM fasudil led to statistically significant increases in cell yield.

Following the observed increase in post-thaw cell recovery, we used flow cytometry to explore the impact of fasudil on the fraction of apoptotic cells over time post-thaw. It was hypothesised, due to the involvement of ROCK in the apoptosis pathway, that its inhibition would decrease the fraction of early apoptotic, then late apoptotic cells. In both 5 and 10% DMSO, initial experiments showed that supplementation of 5 μM and 2.5 μM fasudil post-thaw (respectively) led to increases in the total viable cell population, but the effect was larger in 10% DMSO, matching the recovery data from Fig. 3 (ESI† Fig. S4 and S5). Interestingly, after fasudil supplementation, the fraction of early apoptotic cells decreased most at the earlier post-thaw time points (up to 8 hours) (ESI† Fig. S4 and S5), with minimal differences seen after 24 hours. This is a crucial observation as, by definition, early apoptotic cells will develop into late apoptotic and hence dead/non-viable cells over time. So, whilst taking total cell recovery/viability measurements at 24 hours post thaw is crucial to remove false positives in cryopreservation studies,^25^ the actual measurement of early apoptotic events, must be conducted during this recovery phase. Our results agree with previous literature; in a canine kidney cell line, the percentage of apoptotic cells peaked at 12 hours post-thaw after using multiple cryopreservation solutions, but this number decreased again at the 24 hour time-point.^62^ In bone-marrow mesenchymal stem cell cryopreservation studies, apoptotic cell number peaked (∼20%) at the 2 and 4 hour timepoints, and viability recovered 24 hours post-thaw, possibly due to culture re-population.^63^

However, after performing multiple independent repeats in this study, the overall fraction of cells at each stage of apoptosis did not significantly change after treatment with fasudil, as shown in Fig. 4 (calculated by a Welch *T*-test comparing treated and untreated cells at each time-point). This is probably aggravated by the variability between independent experiments and different cellular state post-thaw in each freezing repeat, as cells might suffer from more post-thaw stress in some replicates than in others. Overall, this does not affect the observation that viable total cell recovery increased compared to cells cryopreserved in 5 or 10% DMSO only (Fig. 3). This method of calculating apoptotic cells using flow cytometry requires the acquisition of a fixed number of events per sample; therefore, it is not designed to calculate total number of viable or apoptotic cells, but to calculate cell population fractions (viable, apoptotic, dead), as supported by previous work.^64^ So, even if more cells were “rescued” at one of the conditions, if the viable/early apoptotic/late apoptotic population fractions did not change then this would not be detected. A study by Baust *et al.* provided comparable results to the current study; where adding an apoptosis inhibitor improved post-thaw cell health, but there was minimal reduction in apoptosis levels. This was attributed to the use of a single caspase inhibitor, rather than a pan-caspase inhibitor.^20^ Additionally, the apoptosis detection technique used in this study is based on identifying cell membrane changes associated with apoptosis.^65^ However, severe loss of membrane integrity, a characteristic of late apoptotic/dead cells, might cause dead cells to be underrepresented, as these fragile cells might be lost during sample washing steps or indistinguishable from debris during flow cytometry analysis.

Reactive oxygen species (ROS) are constantly produced during reduction–oxidation reactions.^66,67^ ROS production is tightly regulated; therefore, any changes can affect a wide range of cellular biological processes. Excessive ROS production (or lack of clearance) can trigger oxidative damage; causing membrane and organelle damage, and disrupting cell signalling pathways, including apoptosis.^67^ The mitochondria are an important source of ROS,^68,69^ so, an excessive production could indicate mitochondrial damage. Similarly, in cryopreservation studies, oxidative stress has been reported to impair mitochondrial functionality^70,71^ and cause intrinsic (mitochondrial) apoptosis.^51^ To probe ROS activity, cells labelled with carboxy-2′,7′-dichlorodihydrofluorescein diacetate (carboxy-H_2_DCFDA) were assessed using flow cytometry. This pro-fluorescent compound exhibits green fluorescence only when it has been oxidised by intracellular ROS, and acts as a general oxidative stress indicator.^72^ It should be noted that ROS are produced during normal cellular metabolism and that the results shown here have been normalised against the negative control.^73^ Jurkat cells cryopreserved in 5 and 10% DMSO and assessed 24 hours post-thaw showed enhanced ROS levels compared to the control (Fig. 5). It is important to highlight that the range of values was broad, with some cells showing extensive oxidative stress compared to others, but the variability was observed between different biological repeats, not technical. For all cases, post-thaw addition of fasudil at 2.5 or 5 μM led to a decrease in the total ROS, but these differences were not statistically significantly different when assessed using the Kruskal–Wallis test with Dunn's *post hoc* test. For the 5% DMSO dataset, the Kruskal Wallis test results reported significantly different populations (*p* = 0.041) however, Dunn's *post hoc* test showed no significant differences between the groups, with only the pairwise comparison of 5% DMSO and control approaching statistical significance (*p* = 0.056). The high variability observed within the data and the overall small sample size (*n* = 3 and *n* = 4) possibly contributed to these observations. Overall, 5% DMSO conditions led to a greater decrease, which could be attributed to the lower DMSO concentration in the freezing media intrinsically leading to less ROS production (rather than the cryopreservation process itself), as an increase in ROS seems to be one of the signs of DMSO toxicity in several cell types^74,75^ These results show that ROS suppression could potentially further improve cell recovery after cryopreservation.

As a final measure of the post-thaw health of fasudil treated cells, growth curves were recorded over 96 hours (Fig. 6). It is important to note that all cells were seeded at equal density at 0 h post-thaw to remove the effects of low post-thaw recovery. The cells cryopreserved in 5 and 10% DMSO with no post-thaw supplementation of fasudil showed slightly delayed growth compared to unfrozen control cells. This difference was especially observed in cells cryopreserved in 5% DMSO. Addition of fasudil for 4 hours post-thaw led in all cases to an increase in growth rate, with higher cell numbers more comparable with the control. In cells cryopreserved with 5% DMSO (Fig. 6A), there were significant differences in cell number between the control and both 5% DMSO and 5% DMSO + 5 μM fasudil (*p* = 0.0046, *p* = 0.041 respectively, 2-way repeated measures ANOVA). In depth pairwise comparisons showed that at the 72 and 96 hours post-thaw time points, the unfrozen control and 5% DMSO conditions showed significant differences in cell number. Thawed cells supplemented with fasudil were not statistically significantly different from the control at any timepoint. In cells cryopreserved with 10% DMSO (Fig. 6B), there was a significant difference between non-supplemented cells and the control (*p* = 0.036, 2-way repeated measures ANOVA). Additional comparisons showed that the unfrozen control and 10% DMSO conditions were statistically significantly different at the 72 h timepoint. This confirms that addressing the biochemical damage pathways not only reduces apoptosis but leads to improved cellular health and robust culturable cells. This measurement is important for example for engineered T-cells, as their therapeutic action requires them to remain viable post-transfusion for as long as possible.

The use of biochemical pathway inhibitors in post-thaw media is appealing but could be technically challenging to achieve in a therapeutic setting (which is beyond the scope of this work), as this would add an additional manufacturing step to the thawed cell product. To address this, we attempted to add fasudil immediately before freezing, without pre-incubation. Then after thawing, the cells were not washed, but diluted with media and their recovery measured after 24 h. This is comparable to a therapeutic setting where thawed cells are injected along with their cryoprotectants. In this case a 20% increase in total cell number was achieved (ESI† Fig. S8A), showing it is in theory possible to create a post-thaw apoptosis modulating one-pot cryopreservation solution.

## Conclusions

Here we demonstrate that the addition of ROCK (Rho-associated-kinases) inhibitors into the thawing medium of a model T-lymphocyte cell line (Jurkats) increases the post-thaw cell yield and recovery. The typical cryopreservation method for Jurkats is the use of DMSO-alone, which is successful but does not enable complete cell recovery, as it only addresses the biophysical, rather than biochemical causes of cell death. Flow cytometry analysis of Jurkats after standard DMSO-only cryopreservation revealed that using 5 or 10% DMSO, there are approximately 20% cells in the early apoptotic stage in the first 8 hours post-thaw, and hence could be addressed by specific biochemical pathway inhibitors. Fasudil (a ROCK inhibitor) was found to give optimum cell recovery when applied to cells in the thawing media during the first 4 and 8 hours post-thaw, with longer exposures decreasing cell yield. Following optimisation, the simple addition of 2.5 μM fasudil after cryopreservation in 10% DMSO led to a 20% increase in cell yields without any need to adjust the cryopreservation process itself. This pattern was also observed after supplementation in 5% DMSO. Flow cytometry analysis showed, in initial studies, a small reduction in apoptosis during the 8 hours after thawing. However, along multiple replicates, this reduction was not significantly different. Despite the variability between repeats, evidence was also found that fasudil reduced reactive oxygen species, supporting the hypothesis that the observed benefit was of biochemical origin. Post-thaw growth curves for up to 96 hours show that fasudil treatment rescued the growth rate compared to standard DMSO cryopreservation, demonstrating that the cells were healthier as well as in greater number. Taken together, this study shows that specifically targeting post-thaw degradative mechanisms can increase the yield and viability of cells following cryopreservation, without adjusting the cryopreservation process itself. This shows that ‘drugging’ cryopreservation, as well as mitigating physical damage due to ice growth is a valid strategy for increasing post-thaw yields of T-cells and may be applicable to T-cell based therapies.

## Associated content

Additional experimental procedures and underpinning data are included in the ESI.†

## Data access statement

Any research data supporting this publication is found in the ESI† or also at https://www.wrap.warwick.ac.uk.

## Author contributions

The manuscript was written through contributions of all authors. All authors have given approval to the final version of the manuscript.

## Conflicts of interest

The authors declare no conflict.

## Supplementary Material

MD-014-D3MD00378G-s001
