# Supplementary material for: Post-thaw application of ROCK-inhibitors increases cryopreserved T-cell yield
Source: RSC Med Chem. 2023 Sep 5;14(10):2058–67. doi: 10.1039/d3md00378g (PMC10583820; doi:10.1039/d3md00378g)
Supplement: MD-014-D3MD00378G-s001 [file MD-014-D3MD00378G-s001.pdf]

Electronic Supplementary Material (ESI) for RSC Medicinal Chemistry.
This journal is © The Royal Society of Chemistry 2023

Supplementary information for

Post-Thaw Application of ROCK-Inhibitors Increases Cryopreserved T-Cell Yield

Natalia Gonzalez-Martinez,[a] and Matthew I. Gibson [a,b]*

[a]Department of Chemistry, University of Warwick, Gibbet Hill Road, Coventry, CV4 7AL, UK

[b]Division of Biomedical Sciences, Warwick Medical School, University of Warwick, Gibbet Hill Road, Coventry, CV4 7AL, UK.

* Corresponding Author m.i.gibson@warwick.ac.uk

Cell culture

Jurkat cells E6.1 (ECACC 88042803) were cultured in 175 cm^2 cell culture flasks (Corning). The cell culture media consisted of Advanced RPMI 1640 media (Gibco) supplemented with 10% foetal bovine serum (FBS) (Sigma Aldrich) and 100 units mL^{-1} penicillin, 100 µg mL^{-1} streptomycin, and 250 ng mL^{-1} amphotericin B (1% Antibiotic-Antimycotic, Gibco). Jurkat cells were maintained at 37 °C and 5% CO_2 in an incubator and maintained at a density between 1.5 and 9 x 10^5 cells mL^{-1}. Cells were passaged every 3-4 days. Mycoplasma tests were regularly performed.

Cell cryopreservation

Cell cryopreservation media consisted of Advanced RPMI 1640 media supplemented with 10% FBS and either 2.5, 5 or 10 % DMSO (Sigma), depending on the experiment performed. Before cryopreservation, Jurkat cells were centrifuged at 300 g and resuspended in antibiotic-free cell culture media at a density of approximately 8 x 10^6 cells mL^{-1}. 500 µL of this cell suspension was pipetted into a 2 mL cryovial (Sigma Aldrich), freezing a total of 4 x 10^6 cells. 500 µL of cryopreservation media (prepared at 2 x the final DMSO concentration, e.g., 10% DMSO for a final concentration of 5%) was then added into the cell suspension. Cryovials were then placed in a Cool LX vial freezing container (Corning) and into a –80 °C freezer, to cool at a rate of 1 °C/min. After 24 hours, cells were thawed in a water bath at 37 °C for 2-3 minutes until only a small ice crystal was left, and the cell suspension was diluted in 9 mL of cell culture media, centrifuged at 300 g for 5 minutes and resuspended in 1 mL cell culture media.

Addition of fasudil hydrochloride into post-thaw media

After resuspending the cryovial contents in 1 mL of cell culture media, 50 µL of cell suspension was placed into each well of round bottom 96 well plates (Sarstedt). Fasudil hydrochloride (Sigma) solutions were then prepared at 2 x the desired concentration in cell culture media and sterile filtered with a 0.22 µm syringe filter (Fisher Scientific**)**. 50 µL was added to each well. The final fasudil concentration used varied from 40 µM to 1.25 µM. Cells were then incubated at 37 °C and 5% CO_2 for 4 h (timepoint selected after performing optimisation experiments). The plate was subsequently centrifuged at 300 g and the media was changed to regular cell culture media. Cell health assessments (such as cell recovery) were performed 24 hours post-thaw. The minimum number of technical replicates per condition was 3.

Cell recovery calculations

24 hours post-thaw, an aliquot of cells was diluted 1:1 with 0.4% trypan blue (Sigma Aldrich). The number of cells with intact membranes (unstained cells) were counted using a haemocytometer (Sigma Aldrich). Cell recovery was calculated by dividing the number of live cells obtained post-thaw against the cell number frozen and expressed as a percentage.

Cytotoxicity assay

Cytotoxicity of fasudil hydrochloride was assessed by measuring the metabolic reduction of resazurin to resorufin in treated and untreated Jurkat cells. 40 000 cells were seeded in 50 µL of cell culture media in a 96 well plate. 2 x solutions of fasudil hydrochloride (Final concentration 40 – 1.25 µM) were prepared in cell culture media, sterile filtered and 50 µL was added to each well, making up a total volume of 100 µL. Cells were placed in an incubator at 37 °C and 5% CO_2. After 24 hours, plates were centrifuged at 300 g for 5 minutes at room temperature and cells were resuspended with 100 µL of resazurin sodium salt (Scientific Laboratory Supplies). The resazurin sodium salt tablet was diluted 1 in 10 in cell culture media. Cells were incubated for 4 h at 37 °C and absorbance was measured using a Synergy HTX Multi-Mode Reader (BioTek) at 570 and 600 nm. Experimental results were expressed as a percentage of absorbance in untreated cells.

Growth curve measurements

Vials frozen with either 5 or 10 % DMSO were thawed, and live cell number was determined by the trypan blue assay as previously described. Then, after the count, 25 000 live cells per well were plated in a round bottom 96-well plate in 100 µL of media. The plates were divided into different conditions (supplemented with fasudil versus untreated) and different timepoints (24-, 48-, 72- and 96-hours post-thaw). Each condition was assessed in triplicates. Depending on the experimental condition, cells were left untreated or treated for 4 hours in 5 or 2.5 µM fasudil hydrochloride, after being frozen in 5 and 10 % DMSO respectively. After 72 hours, cells at all conditions were supplemented with an additional 100 µL of cell culture media. Cell number was determined using the trypan blue assay at each timepoint.

Flow cytometry

Flow cytometry was performed using a BD Accuri C6 Plus flow cytometer (BD Biosciences). 20,000 events were acquired per sample for all experiments. CS&T Research beads (BD Biosciences) were used for instrumental quality control before performing experiments. During

the experiments, the flow rate used was 35 μL/min (16 μm core). Green (FITC/H_2DCFDA) fluorescence was acquired using the 488 nm excitation laser and 530/30 nm emission filter. Red (Propidium Iodide) fluorescence was acquired by using the 488 nm excitation laser and either the 585/40 or 670 LP emission filters. FlowJo (Version 10) was used to analyse and plot flow cytometry data. Fluorescence compensation was manually performed to minimise overlap of the FITC (FL-1) and PI (FL-2, FL-3) channels using fluorescence minus one (FMO) control from experimental positive and negative controls.

Reactive Oxygen Species (ROS) assessment

ROS measurements were performed 24 hours after thawing the cells, and, either after leaving them untreated, or treating them for 4 h with 2.5 and 5 μM fasudil hydrochloride. Cells were then plated into 24 well plates (Falcon) and washed twice in DPBS (Dulbecco's phosphate-buffered saline) prior to loading with the ROS detection dye to avoid extracellular hydrolysis by acetoxymethyl and acetate esters as outlined in the product protocol. The ROS detection dye consisted of 1 μM of carboxy-H_2DCFDA (Thermo Fisher) in DPBS, and it was loaded into the cells for 30 minutes at 37 °C and 5 % CO_2. Subsequently, cells were washed in DPBS and returned to the incubator at 37 °C and 5 % CO_2 for 5 minutes to allow cells to recover after the staining procedure. Samples were then analysed using flow cytometry as described in the above section. The negative control consisted of unfrozen stained cells. Positive controls consisted of unfrozen stained cells incubated with 125, 250 or 500 μM H_2O_2 for 1 h at 37 °C and 5 % CO_2 to induce ROS. During the data analysis, the median fluorescence intensity was extracted from each sample analysed by flow cytometry using FlowJo. Experimental results were normalised relative to (i.e., divided by) the mean of the (median) fluorescence values from the negative control.

Apoptosis analysis

Cryovials of cells frozen in either 2.5, 5 % or 10 % DMSO at 4 x 10^6 cells mL^{-1} were thawed in a water bath at 37 °C for 2 minutes. Thawed cells were placed into 12 well plates (Falcon) at a concentration of approximately 500 000 cells per well in triplicates for their use at each experimental timepoint. To treat cells with fasudil post-thaw, after thawing the vial and centrifuging cells, cells were resuspended with the desired concentration of fasudil (2.5 or 5 μM) and incubated for 4 h post-thaw. After this time, cells were resuspended in cell culture media for future analysis (relevant for the 8 and 24 h timepoints). Additionally, unfrozen, untreated cells were plated at the same density to act as a negative control of apoptosis. A

positive control was created by treating cells with the apoptosis inducer staurosporine (APExBio) at a 1 μM concentration, this incubation started 2 h before analysis of the first timepoint (at 0 h post-thaw). After 2, 4, 8 and 24 hours, cell suspensions were removed from the incubator and were prepared for flow cytometry analysis of apoptosis. The FITC Annexin V/PI apoptosis kit for flow cytometry (Fisher Scientific) was used, and samples were prepared following manufacturer instructions. Cells were then incubated at room temperature in the dark for 15 minutes and kept in ice until analysed using flow cytometry as described in the flow cytometry section.

The assay used is based on the known cellular externalisation of phosphatidylserine – a phospholipid found in the inner membrane- to the outer membrane during apoptosis. Annexin V can then bind phosphatidylserine in presence of calcium ions but cannot bind other phospholipids. This translocation can be observed from early phases of apoptosis, where the plasma membrane has not yet ruptured, therefore apoptotic and other sources of cell death such as necrosis can be distinguished. On later stages of apoptosis, cells undergo secondary necrosis, where the membrane is damaged and allows internalisation of propidium iodide (PI), normally excluded from the cell membrane, that upon binding to DNA in the cell displays orange/red fluorescence.[1]

Performing the analysis at several timepoints post-thaw allows improved accuracy of distinction between different modes of cell death, as it is necessary to ensure cells are undergoing early apoptosis (by staining positive for Annexin V only) before reaching late apoptosis/cell death (both stains). If the assay was solely performed at late timepoints such as 24 hours post-thaw, it would be difficult to distinguish cells undergoing apoptosis and necrosis.

Dual colour fluorescence density plots were created to identify the cell health of the population. Unstained samples (Q4) were classified as "viable", cells stained only for Annexin V (green fluorescence) were defined as "early apoptotic" (Q3) and cells staining positive for PI as "late apoptotic/dead" (Q2).

Fasudil supplementation in cryopreservation media experiments

Two different cryopreservation media were compared in this section: Advanced RPMI 1640 media supplemented with 10 % FBS and 5 % DMSO, and that same media supplemented with 50 μM fasudil hydrochloride. DMSO and fasudil were prepared at 2 x the concentration, sterile filtered, and kept at 4 °C until use. Jurkat cells were harvested, counted, and resuspended in Advanced RPMI 1640 media supplemented with 10 % FBS. The target cell number was 10 x

10^6 cells mL^{-1}. 500 µL of this cell suspension (5 x 10^6 cells approx.) were then placed into cryovials (Nalgene) followed by 500 µL of the cryopreservation media. Cryovials were then placed in a Cool LX vial freezing container and into a –80 °C freezer. After 24 hours, cryovials were thawed for 2-3 minutes in a water bath at 37 °C until no ice crystals were visible, and the 1 mL cell suspension was transferred into a 15 mL Falcon tube containing 9 mL of cell culture media. The tube contents were gently mixed and 100 µL of cell suspension were plated into round bottom 96-well plates (Starstedt), targeting 50 000 cells per well without considering cryopreservation damage. Unfrozen cells were plated at this cell density as a control for metabolic activity assays. For cell recovery studies, cell density before cryopreservation was used to calculate percentage recovery. All cell health assays were performed 24 hours post-thaw.

Statistical analysis

Statistical analysis was performed using Origin (Version 2022). The data was analysed for normality using the Shapiro-Wilk Test, and Levene Test to test for equality of variance between groups. When the distribution and variances were equal between groups, mean comparisons among two or more groups were performed using one-way ANOVA and Tukey's post-hoc test against the appropriate control and results were reported as mean ± SD, as shown in Figures 1, 2 and 3. For growth curve experiments, a 2-way repeated measures ANOVA was performed to compare the effect of treatment (or its absence) and time post-thaw, on cell number (Figure 6). Results were considered statistically significantly different when $p < 0.05$. When the tests determined a significant difference between the distribution, or equality of variance tests, a non-parametric test (Kruskal-Wallis) was used, together with Dunn post-hoc test (Figure 5). Results were visually represented using boxplots, highlighting the median and upper and lower quartiles. Additionally, the Welch T-test was used for non-parametric comparison between 2 groups (Figure 4).

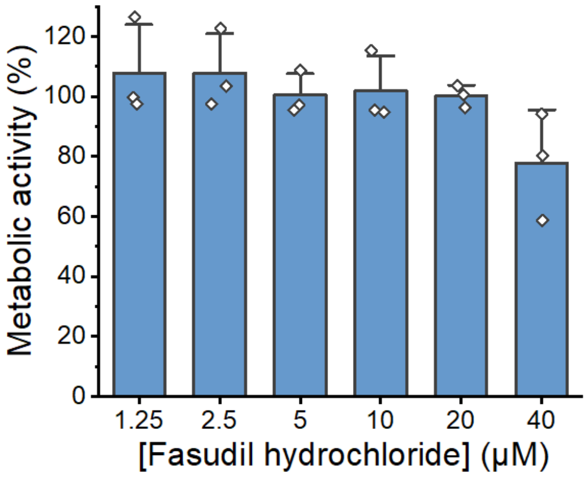

Figure 1. Metabolic activity of Jurkat cells after a 24-hour incubation period with a range of fasudil hydrochloride concentrations. Metabolic activity (%) was measured using the resazurin reduction assay and reported relative to untreated cells. Results expressed as the mean ± SD of 3 independent experiments, each formed of 6 technical replicates.

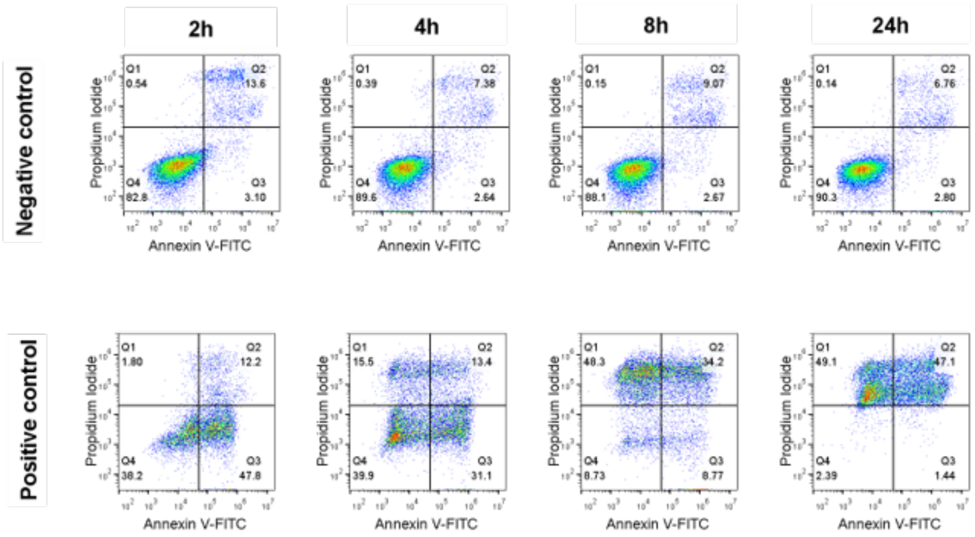

Figure 2. Representative density dot plots of negative and positive controls for apoptosis analysis over a 24-hour time-period. Staining was performed using Annexin V-FITC and Propidium Iodide kit (Thermo Fisher) following manufacturer instructions. The negative control consisted of fresh, untreated, stained cells. The positive control consisted of cells incubated with 1 µM staurosporine for 1 h prior to the start of experiment. Viable cells are shown as Annexin V^-, PI^-. Early apoptotic cells are shown as Annexin V^+, PI^-, Late apoptotic/dead cells shown as Annexin V^+, PI^+ or Annexin V^+, PI^-. 20 000 cells were acquired

per sample. FITC fluorescence was detected using the 488 nm excitation laser and 530/30 nm emission filter, and PI fluorescence was detected using the 488 nm excitation laser and 670 nm LP emission filter with a BD Accuri C6 Plus flow cytometer.

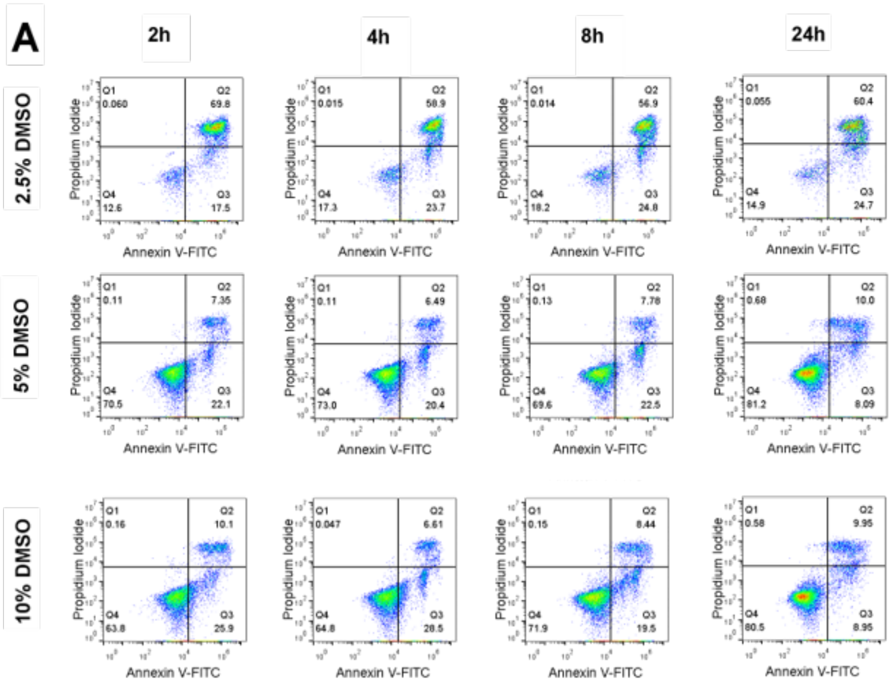

Figure 3 Representative apoptosis analysis density dot plots for cells cryopreserved in media containing 2.5-10% DMSO. Staining was performed using Annexin V-FITC and Propidium Iodide kit (Thermo Fisher) following manufacturer instructions. Viable cells shown as Annexin V^-, PI^-. Early apoptotic cells are shown as Annexin V^+, PI^-, Late apoptotic/dead cells shown as Annexin V^+, PI^+ or Annexin V^+, PI^-. 20 000 cells were analysed per sample. FITC fluorescence was detected using the 488 nm excitation laser and 530/30 nm emission filter, and PI fluorescence was detected using the 488 nm excitation laser and 670 nm LP emission filter with a BD Accuri C6 Plus flow cytometer.

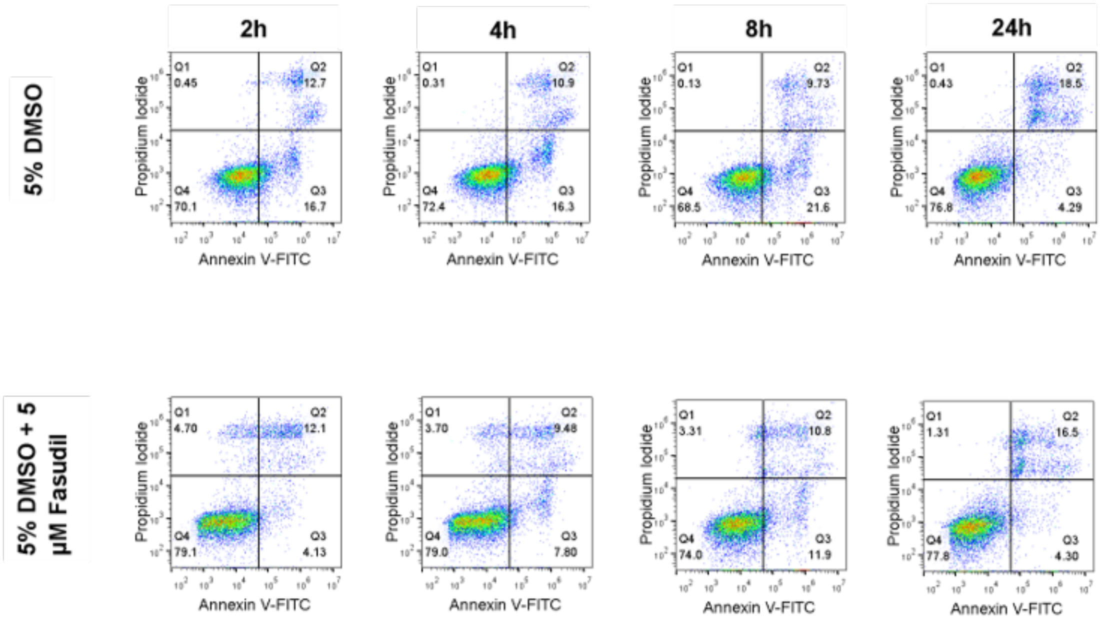

Figure 4. Representative density dot plots of apoptosis analysis for cells cryopreserved in 5% DMSO. Cells were then either left untreated or supplemented with 5 µM of fasudil hydrochloride post-thaw for 4 h. Staining was performed using Annexin V-FITC and Propidium Iodide kit (Thermo Fisher) following manufacturer instructions. Viable cells shown as Annexin V$^-$, PI$^-$. Early apoptotic cells are shown as Annexin V$^+$, PI$^-$, Late apoptotic/dead cells shown as Annexin V$^+$, PI$^+$ or Annexin V$^+$, PI$^-$. 20 000 cells were analysed per sample. FITC fluorescence was detected using the 488 nm excitation laser and 530/30 nm emission filter, and PI fluorescence was detected using the 488 nm excitation laser and 670 nm LP emission filter with a BD Accuri C6 Plus flow cytometer.

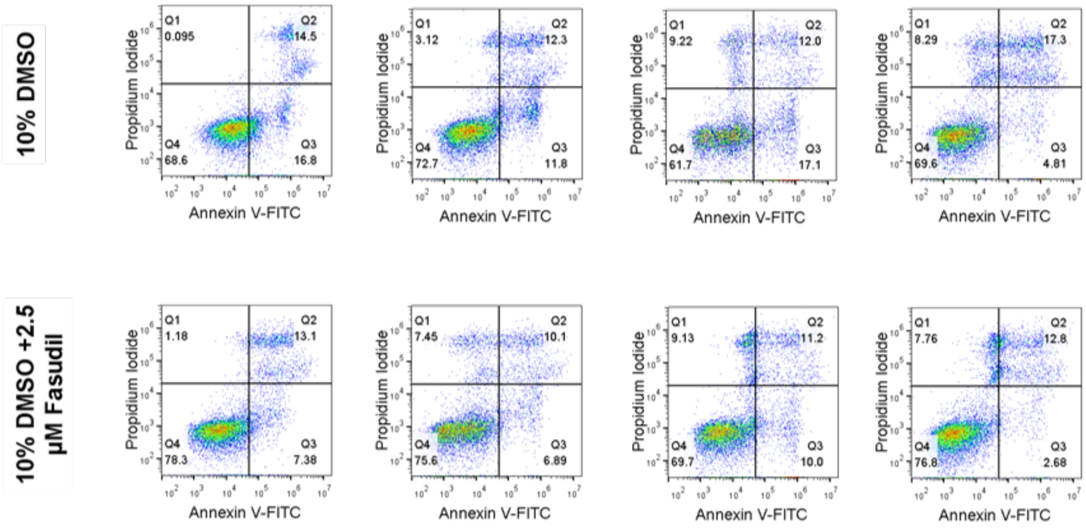

Figure 5. Representative density dot plots of apoptosis analysis for cells cryopreserved in 10% DMSO. Cells were then either left untreated or supplemented with 2.5 µM of fasudil hydrochloride post-thaw for 4 h. Staining was performed using Annexin V-FITC and Propidium Iodide kit (ThermoFisher) following manufacturer instructions. Viable cells shown as Annexin V^-, PI^-. Early apoptotic cells are shown as Annexin V^+, PI^-, Late apoptotic/dead cells shown as Annexin V^+, PI^+ or Annexin V^+, PI^-. 20 000 cells were analysed per sample.

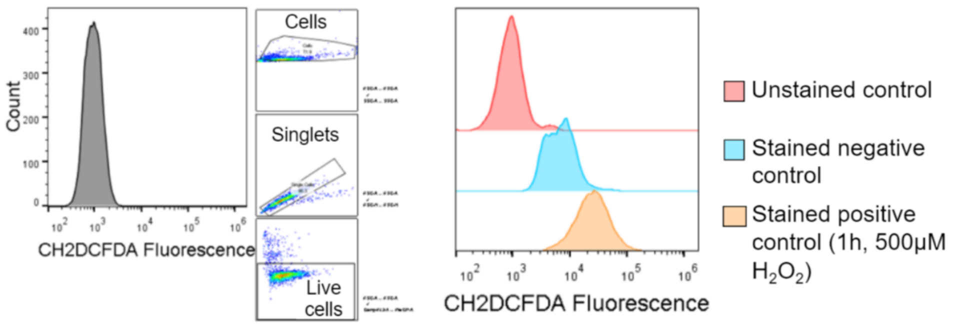

Figure 6. Overview of the gating strategy and experimental controls used during the ROS experiments. Regarding the gating strategy, debris were excluded by size using FSC-A/SSC-A (FSC = forward scatter, SSC = side scatter). To ensure single cell analysis, doublets were excluded by gating using FSC-A vs FSC-H (forward scatter area vs height). Propidium iodide (PI) staining (final concentration 1 µg mL^{-1}) was used to determine live/dead cells. PI fluorescence was detected using the 488 nm excitation laser and 670 nm LP emission filter. Live cells were gated as PI negative. This ensures only viable cells are included in the subsequent analyses. In subsequent analyses samples were stained with carboxy-H_2DCFDA and fluorescence was detected using the 488 nm excitation laser and 530/30 nm emission filter with a BD Accuri C6 Plus flow cytometer. The controls used were an unstained, unfrozen control, stained negative control, using fresh cells in culture and a stained positive control, with cells treated with 500 µM H_2O_2 for 1 h to induce ROS production.

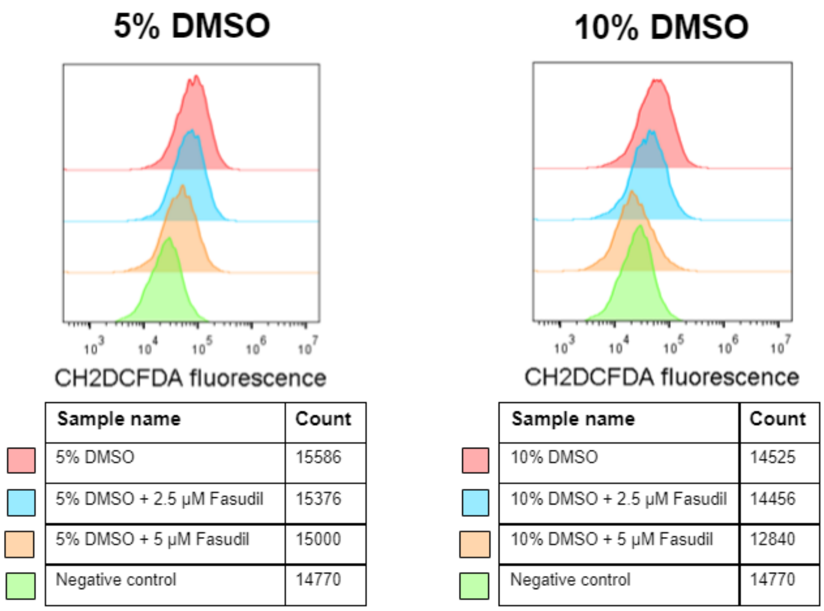

Figure 7. Carboxy-H_2DCFDA fluorescence histograms for ROS detection. ROS analysis was performed 24 h after thawing cells cryopreserved in either 5 % or 10 % DMSO. After thawing, cells were either left untreated or supplemented with Fasudil for 4 hours, then analysed after 24 hours. Fluorescence was detected using the 488 nm excitation laser and 530/30 nm emission filter with a BD Accuri C6 Plus flow cytometer. The negative control consisted of stained, unfrozen cells in culture.

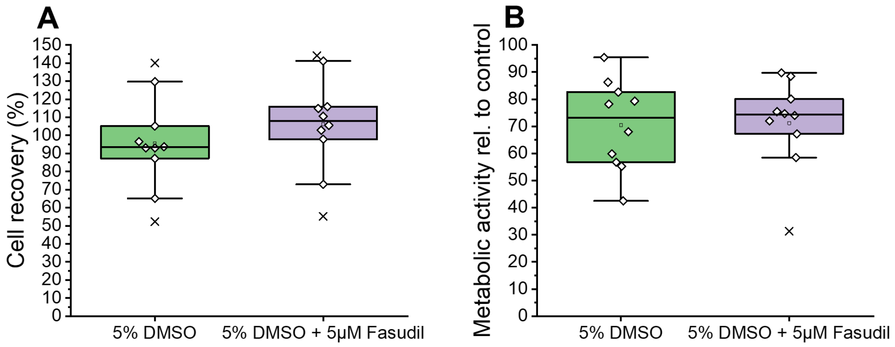

Figure 8. Post-thaw evaluation of the addition of 50 μM fasudil hydrochloride into cryopreservation media supplemented with 5% DMSO. (A) Cell recovery 24 hours post-thaw calculated using trypan blue assay. (B) Metabolic activity 24 hours post-thaw using resazurin reduction assay. Each datapoint represents the mean of an independent experiment, each with > 3 technical replicates. The top and bottom ends of the boxplot represent the upper and lower quartile, line represents the median of the dataset and the square represents the mean, whiskers

denote the upper and lower points of the dataset (within 1.5 x IQR), and the crosses indicate outliers.

References

1 B. H. Villas, *Cell Vis.*, 1998, **5**, 56–61.
